# Dynamic surveillance of lymphocyte subsets in patients with non-small cell lung cancer during chemotherapy or combination immunotherapy for early prediction of efficacy

**DOI:** 10.3389/fimmu.2024.1316778

**Published:** 2024-02-28

**Authors:** Shanshan Zhen, Wenqian Wang, Guohui Qin, Taiying Lu, Li Yang, Yi Zhang

**Affiliations:** ^1^ Biotherapy Center, the First Affiliated Hospital of Zhengzhou University, Zhengzhou, Henan, China; ^2^ Department of Oncology, the First Affiliated Hospital of Zhengzhou University, Zhengzhou, Henan, China; ^3^ School of Life Sciences, Zhengzhou University, Zhengzhou, Henan, China; ^4^ State Key Laboratory of Esophageal Cancer Prevention & Treatment, Zhengzhou University, Zhengzhou, Henan, China

**Keywords:** lymphocyte subsets, immunotherapy, chemotherapy, efficacy prediction, NSCLC

## Abstract

**Background:**

Non-small cell lung cancer (NSCLC) remains the leading cause of cancer-related deaths worldwide. Lymphocytes are the primary executors of the immune system and play essential roles in tumorigenesis and development. We investigated the dynamic changes in peripheral blood lymphocyte subsets to predict the efficacy of chemotherapy or combination immunotherapy in NSCLC.

**Methods:**

This retrospective study collected data from 81 patients with NSCLC who received treatments at the First Affiliated Hospital of Zhengzhou University from May 2021 to May 2023. Patients were divided into response and non-response groups, chemotherapy and combination immunotherapy groups, and first-line and multiline groups. We analyzed the absolute counts of each lymphocyte subset in the peripheral blood at baseline and after each treatment cycle. Within-group and between-group differences were analyzed using paired Wilcoxon signed-rank and Mann-Whitney U tests, respectively. The ability of lymphocyte subsets to predict treatment efficacy was analyzed using receiver operating characteristic curve and logistic regression.

**Results:**

The absolute counts of lymphocyte subsets in the response group significantly increased after the first cycle of chemotherapy or combination immunotherapy, whereas those in the non-response group showed persistent decreases. Ratios of lymphocyte subsets after the first treatment cycle to those at baseline were able to predict treatment efficacy early. Combination immunotherapy could increase lymphocyte counts compared to chemotherapy alone. In addition, patients with NSCLC receiving chemotherapy or combination immunotherapy for the first time mainly presented with elevated lymphocyte levels, whereas multiline patients showed continuous reductions.

**Conclusion:**

Dynamic surveillance of lymphocyte subsets could reflect a more actual immune status and predict efficacy early. Combination immunotherapy protected lymphocyte levels from rapid decrease and patients undergoing multiline treatments were more prone to lymphopenia than those receiving first-line treatment. This study provides a reference for the early prediction of the efficacy of clinical tumor treatment for timely combination of immunotherapy or the improvement of immune status.

## 1 Introduction

Lung cancer is the most common cancer worldwide and the leading cause of cancer-related deaths (1). Non-small cell lung cancer (NSCLC) accounts for approximately 85% of all lung cancer cases (2). Although various clinical treatments, including chemotherapy, radiotherapy, and targeted therapy, have prolonged the survival of lung cancer patients, the five-year survival outcome of NSCLC remains unsatisfactory (3, 4). With the development of the tumor surveillance theory and continuous researches on the tumor immunity, scientists have increasingly realized the essential roles of the immune system in tumor control. The advent of immunotherapy has profoundly revolutionized cancer treatment because of its continuous therapeutic effects brought by immune memory (5–7). As the fourth modality of modern tumor treatment, immunotherapy, which controls tumors by mobilizing the immune system, is the only treatment that promises to eliminate tumor cells completely (8). The clinical effectiveness of anti-PD-1/PD-L1 therapy demonstrates the vital roles of the immune system in anti-tumor effects (9, 10). Immune status is closely related to tumorigenesis, progression and prognosis (11). Therefore, evaluating immune status of patients is of great significance in the clinical cancer treatment.

Classical lymphocyte subsets are classified into T cell, including CD4^+^ T cell and CD8^+^ T cell, B cell and natural killer (NK) cell. Abundant and active lymphocytes are important tumor resistant (8, 12). They are involved in innate and adaptive immunity and work together to exert anti-tumor effects (13). However, in clinical practice, assessing immune status by detecting tumor-infiltrating lymphocytes is not feasible for many patients with advanced cancer because of the difficulty in repeatedly obtaining tumor tissue. The use of easily available peripheral blood is less invasive and more convenient for clinical applications. Increasing evidence suggests that the absolute counts of peripheral blood lymphocytes are positively correlated with tumor prognosis and outcomes (14–16).

Currently, chemotherapy remains a vital treatment option for advanced NSCLC (17). Substances released during chemotherapy-induced tumor cell death may promote lymphocyte activation and proliferation, which are synergistically involved in tumor killing (18). However, long-term chemotherapy can lead to severe lymphopenia. The cytotoxicity of chemotherapeutic drugs and severe myelosuppression caused by chemotherapy affect the production and differentiation of lymphocytes (19). A low-lymphocyte environment affects tumor surveillance and killing, resulting in a highly susceptibility to the failure of tumor control. When there is an inadequate number of effective lymphocytes, the combination of anti-PD-1/PD-L1 therapy may not benefit cancer patients (20). Therefore, detecting dynamic changes in lymphocyte subsets is of great significance for early efficacy prediction, decisions on the replacement of ineffective treatments, the timely utilization of immunotherapy and the timely application of lymphocyte-improving drugs, such as thymosin in the clinic (21, 22).

The aim of this study was to explore the association between efficacies of chemotherapy or chemo-immunotherapy and the absolute counts of lymphocyte subsets in peripheral blood, and we expected to predict the efficacy in advance in order to provide a clinical reference. We also explored the effects of combined immunotherapy on lymphocyte counts. Besides, we noted significant differences in dynamic changes of lymphocyte subsets between patients receiving first-line and multiline treatments.

## 2 Methods

### 2.1 Clinical data collection

We collected data from NSCLC patients who received treatment at the First Affiliated Hospital of Zhengzhou University from May 2021 to May 2023. Eighty-one patients receiving first-line or multiline therapy with standard chemotherapy or chemoimmunotherapy were included in our study. Clinical and pathological data of all patients were collected, including age; sex; smoking history; pathological information; lymphocyte subsets; and imaging findings, such as chest computed tomography (CT) and head magnetic resonance imaging (MRI). Baseline was collected before patients received their first treatment. Collection of lymphocyte subsets each cycle was before the next treatment (three weeks later). This study was approved by the Medical Ethics Committee of the First Affiliated Hospital of Zhengzhou University (2021-KY-1105-002).

### 2.2 Inclusion criteria

1. 18 to 80 years of age
2. Definite pathological diagnosis of non-small cell lung cancer
3. Eastern Cooperative Oncology Group score 0-1, expected survival > 6 months
4. No combination of other tumors, acute infections, blood system diseases, or immune system diseases
5. Received four consecutive cycles of chemotherapy or chemotherapy combined with anti-PD-1 therapy
6. Treated for the first time after diagnosis or treatment again after at least second-line failure

### 2.3 Exclusion criteria

1. Inability to trace personal or clinical date
2. Not followed up, lack of regular treatments or reviews, or inability to assess disease progression
3. The use of immunomodulators, such as thymopeptides or placental polypeptides, during treatment

### 2.4 Group design

1. First-line group: patients who received standard first-line therapy for the first time after diagnosis
2. Multiline group: patients who received retreatment after experiencing at least second-line failure
3. Chemotherapy group: patients who received a chemotherapy regimen during the four treatment cycles
4. Combination group: patients who received anti-PD-1 therapy in combination with four chemotherapy cycles

### 2.5 Efficacy evaluation

A comprehensive assessment of treatment efficacy after four treatment cycles was performed based on CT, MRI, bone scan, and other imaging methods. Complete response (CR), partial response (PR), stable disease (SD), and progressive disease (PD) were determined according to the Response Evaluation Criteria in Solid Tumors 1.1 criteria.

Response group: CR + PR + SD

Non-response group: PD

### 2.6 Statistical analysis

Differences in each basic characteristic between response and non-response groups were analyzed using Chi-square test. Dynamic changes in each lymphocyte subset within the groups in four treatment cycles were subjected to the paired Wilcoxon signed-rank test. The Mann-Whitney U test was used to analyze between-group differences. The receiver operator characteristic (ROC) curve was used to evaluate predictive capacity of lymphocyte subsets and choose the best cut-off values. Cut-off values were determined by calculating the Youden’s Index = Sensitivity + Specificity-1. Combination indicators of two lymphocyte subsets for efficacy prediction as well as model evaluation were analyzed by binary logistic regression with SPSSPRO. Statistical analyses were performed using SPSS 22.0 (IBM, Armonk, NY, USA), and Prism 8.4.3 (GraphPad, San Diego, CA, USA) was used to construct figures.

## 3 Results

### 3.1 Patients’ characteristics

A total of 81 patients were enrolled in this study, including 56 males and 25 females, with a mean age of 61 years old. Forty-eight of the patients had a history of smoking. According to the 8th edition of the American Joint Committee on Cancer staging criteria, 5 patients were in stage I, 9 patients were in stage II, 20 patients were in stage III, and 47 patients were in stage IV. Forty-two patients had identified gene mutation. In addition, immunohistochemistry showed KI67 expression. The expression of KI67 varied from 2 to 90 percent. All medical histories and pathological features were shown in **Table 1**. These characteristics were not significantly different between the response and non-response groups, according to the Chi-squared test (*P* > 0.05). In this study, 45 patients received first-line treatment and 36 patients received multiline treatment. The response rate was significantly higher in the first-line treatment group than the multiline treatment group (*P* = 0.033). Forty-seven patients were treated with chemotherapy alone and 34 received chemotherapy combined with immunotherapy. The response rates of the two treatments were not significantly different (*P* = 0.752).

**Table 1:** The clinical and pathological characteristics of included 81 patients.

Characteristics	Case	Response	Non-response	*χ2*	*P* value
Age (years)				0.105	0.745
≤ 60	39 (48.1%)	30 (37.0%)	9 (11.1%)		
> 60	42 (51.9%)	31 (38.3%)	11 (13.6%)		
Sex				0.428	0.513
Male	56 (69.1%)	41 (50.6%)	15 (18.5%)		
Female	25 (30.9%)	20 (24.7%)	5 (6.2%)		
Smoking history				2.726	0.099
Yes	48 (59.3%)	33 (40.7%)	15 (18.5%)		
No	33 (40.7%)	28 (34.6%)	5 (6.2%)		
Histological type				0.199	1
AD	48 (59.3%)	36 (44.4%)	12 (14.8%)		
SQCC	27 (33.3%)	20 (24.7%)	7 (8.6%)		
Others	6 (7.4%)	5 (6.2%)	1 (1.2%)		
Clinical stage				2.008	0.565
I	5 (6.2%)	5 (6.2%)	0 (0%)		
II	9 (11.1%)	7 (8.6%)	2 (2.5%)		
III	20 (24.7%)	16 (19.8%)	4 (4.9%)		
IV	47 (58.0%)	33 (40.7%)	14 (17.3%)		
Gene mutation				1.74	0.419
Yes	42 (51.9%)	33 (40.7%)	9 (11.1%)		
No	12 (14.8%)	10 (12.3%)	2 (2.5%)		
Unknown	27 (33.3%)	18 (22.2%)	9 (11.1%)		
KI67 (%)				4.925	0.155
2 ≤ KI67 < 30	24 (29.6%)	21 (25.9%)	3 (3.7%)		
30 ≤ KI67 < 80	39 (48.1%)	25 (30.9%)	14 (17.3%)		
80 ≤ KI67 < 90	15 (18.5%)	12 (14.8%)	3 (3.7%)		
Unknown	3 (3.7%)	3 (3.7%)	0 (0%)		
Treatment					
First-line	45 (55.6%)	38 (46.9%)	7(8.6%)	4.545	0.033
Multiline	36 (44.4%)	23 (28.4%)	13(16.0%)		
Chemotherapy	47 (58.0%)	36 (44.4%)	11(13.6%)	0.1	0.752
Combination	34 (42.0%)	25 (30.9%)	9(11.1%)		

AD, adenocarcinoma; SQCC, squamous cell carcinoma. The numbers and percentages of each characteristic in the response and non-response groups were shown. Differences in each characteristic between the two groups were analyzed using the Chi-square Test, and P < 0.05 was considered statistically different.

### 3.2 Relationship between efficacy and dynamic changes in lymphocyte subsets

#### 3.2.1 Significant differences in dynamic changes of lymphocyte subsets between the response and non-response groups

**Figure 1:**
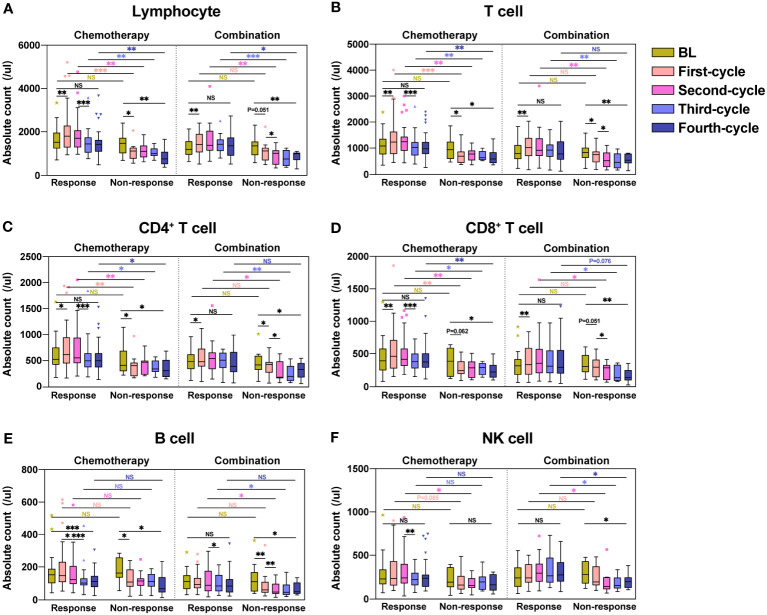
Dynamic changes in lymphocyte subsets during four treatment cycles in the response and non-response groups. Absolute counts of Lymphocyte **(A)**, T cell **(B)**, CD4^+^ T cell **(C)**, CD8^+^ T cell **(D)**, B cell **(E)**, and NK cell **(F)** in the response and non-response groups during four consecutive treatment cycles were shown as boxplots. The chemotherapy and combination immunotherapy groups were shown respectively. Within-group and between-group differences were analyzed using the paired Wilcoxon signed-rank test and Mann-Whitney U test, respectively. BL, Baseline; *, P < 0.05; **, P < 0.01; ***, P < 0.001; NS, not significant.

We respectively analyzed the dynamic changes in lymphocyte subsets in the response and non-response groups of patients who received chemotherapy or combination immunotherapy during four consecutive treatment cycles. Box plots were used to display medians and interquartile ranges of lymphocyte subset counts in each treatment cycle. Significant differences within and between groups were analyzed and marked (**Figure 1**).

After the first chemotherapy cycle, the absolute counts of Lymphocyte (**Figure 1A**, *P* = 0.006), T cell (**Figure 1B**, *P* = 0.004), CD4^+^ T cell (**Figure 1C**, *P* = 0.012) and CD8^+^ T cell (**Figure 1D**, *P* = 0.004) showed significant increases in the response group, with B cell (**Figure 1E**, *P* = 0.134) and NK cell (**Figure 1F**, *P* = 0.177) showing numerical increases, in contrast to the remarkable decreases in the non-response group. The absolute counts of Lymphocyte (**Figure 1A**, *P* = 0.041), T cell (**Figure 1B**, *P* = 0.021), CD4^+^ T cell (**Figure 1C**, *P* = 0.013), and B cell (**Figure 1E**, *P* = 0.037) significantly decreased in the non-response group after the first chemotherapy cycle. CD8^+^ T cell (**Figure 1D**, *P* = 0.062) and NK cell (**Figure 1F**, *P* = 0.155) likewise decreased, although the difference was not statistically significant. Lymphocyte subsets in patients receiving the first combination immunotherapy cycle showed similar trends to chemotherapy in both response and non-response groups. During the four treatment cycles in the response group, lymphocyte subsets in patients receiving chemotherapy alone showed a trend of increasing counts first and then decreasing counts, whereas patients treated with combination immunotherapy showed an increase, followed by a maintenance of high lymphocyte counts. Except for B cell (**Figure 1E**, *P* = 0.001) in the chemotherapy group, lymphocyte subsets in the response group after four treatment cycles were not significantly different from those at baseline. Patients in the non-response group showed significant reduction in the counts of all lymphocyte subsets compared to baseline after four treatment cycles, regardless of whether they received chemotherapy or combination immunotherapy. Of note, the counts of lymphocyte subsets between the response and non-response groups were not significantly different at baseline, but significant differences were observed immediately after treatment. The counts of Lymphocyte (**Figure 1A**), especially T cell (**Figure 1B**), including CD4^+^ T cell (**Figure 1C**) and CD8^+^ T cells (**Figure 1D**), was significantly higher in the response group than those in the non-response group during chemotherapy or combination immunotherapy. B cell (**Figure 1E**) and NK cell (**Figure 1F**) in the response group also showed higher counts compared with the non-response group.

Overall, we found that lymphocyte subset reactions during treatment were strongly associated with the four-cycle treatment efficacy in patients with NSCLC who received chemotherapy or combination immunotherapy.

#### 3.2.2 Predictive value of lymphocyte subsets on the efficacy in NSCLC patients

**Figure 2:**
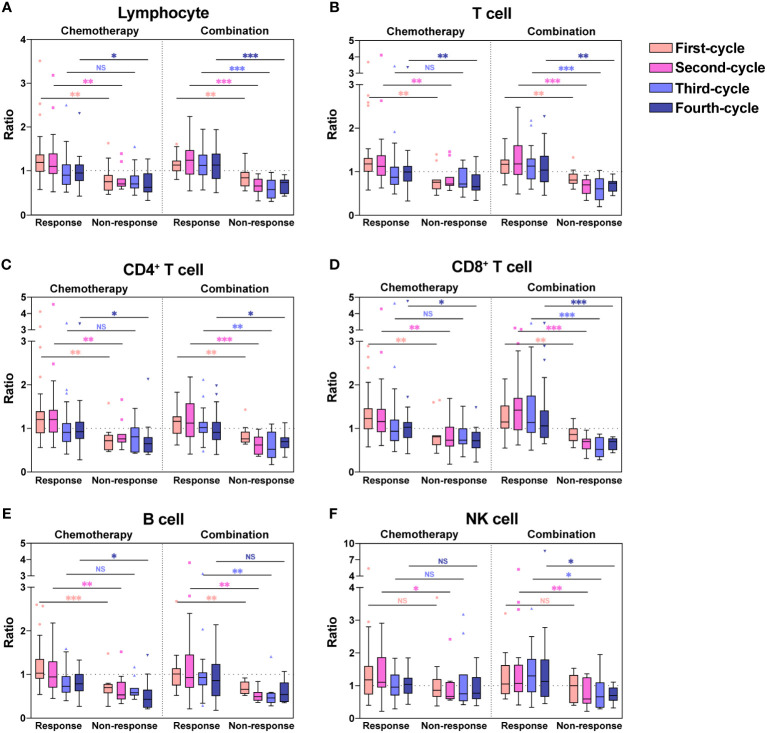
Ratios of absolute counts after each treatment to baseline for each lymphocyte subset. Ratios of absolute counts after each treatment cycle to baseline for Lymphocyte **(A)**, T cell **(B)**, CD4^+^ T cell **(C)**, CD8^+^ T cell **(D)**, B cell **(E)**, and NK cell **(F)** were shown as boxplots. The chemotherapy and combination immunotherapy groups were shown respectively. Between-group differences were analyzed statistically by Mann-Whitney U test. *, P < 0.05; **, P < 0.01; ***, P < 0.001; NS, not significant.

As shown in **Figure 1**, we found significant differences in lymphocyte subsets between the response and non-response groups. To better demonstrate the changes in lymphocytes, we used box plots to show the ratios of lymphocyte subset count after each treatment cycle to the counts at baseline (**Figure 2**). The ratios of each lymphocyte subset in the response group were almost greater than 1, especially in the first two chemotherapy or combination cycles, in contrast to the non-response group, in which the ratios were less than 1 throughout the treatment cycles. The ratios of Lymphocyte (**Figure 2A**), T cell (**Figure 2B**), CD4^+^ T cell (**Figure 2C**), CD8^+^ T cell (**Figure 2D**) and B cell (**Figure 2E**) were significantly higher in the response group than the non-response group (*P* < 0.05). Likewise, the ratios of NK cell were higher in the response group than the non-response group in some of the treatment cycles (**Figure 2F**, *P* < 0.05). Regardless of whether patients received chemotherapy or combination immunotherapy, they showed similar differences in the ratios between the response and non-response groups. Owing to the cytotoxicity of chemotherapy, patients receiving chemotherapy alone in the response group experienced significant lymphopenia after the third chemotherapy cycle, leading to no significant difference in ratios between the response and non-response groups after the third chemotherapy cycle compared to baseline (**Figures 2A-E**, *P* > 0.05). While the protective effect of combination immunotherapy on lymphocytes reduced lymphopenia caused by long-term chemotherapy (**Figures 2A–E**).

**Figure 3:**
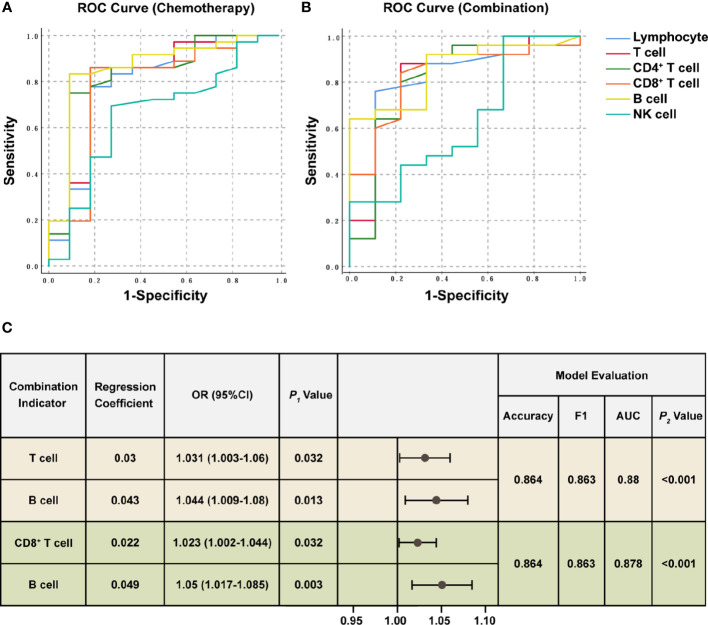
ROC analysis and Logistic regression of lymphocyte subsets for efficacy prediction. ROC curves were plotted for the ratios of each lymphocyte subset counts after the first treatment cycle to baseline in the response and non-response groups. The chemotherapy **(A)** and combination immunotherapy **(B)** groups were shown, respectively. AUC, significance, asymptotic 95% confidence interval, and cut-off values were shown in **Table 2**. **(C)** Logistic regression analysis of combined lymphocyte subsets for efficacy prediction. The two sets of combination indicators with the best predictive power and model evaluation were displayed. P_1_ value represented the significance of each lymphocyte subset for efficacy prediction. Model evaluations were generated from logistic regression. F1 score combines the precision and recall to measure accuracy. ROC curves and AUC were used to measure the classification capacity of logistic regression. P_2_ value was the likelihood ratio chi-square test to evaluate the validity of predictive models. P < 0.05 was considered statistically significant. OR: odds ratio, 95%CI: 95% confidence interval.

The ratios were significantly higher in the response group than the non-response group for all chemotherapy cycles. Based on our previous analysis, lymphocyte levels in the response group and non-response groups did not demonstrate significant differences at baseline, whereas the ratios of lymphocyte count after the first chemotherapy cycle to baseline were significantly higher in the response group. This suggested that the response group possessed a more dynamic and more easily activated immune environment. Therefore, we hypothesized that the lymphocyte ratios after the first treatment cycle to baseline could predict the four-cycle treatment efficacy in patients with NSCLC. We analyzed the ROC curves in the response and non-response groups, and patients receiving chemotherapy alone or combination immunotherapy were analyzed separately (**Figures 3A, B**). The areas under the curve (AUCs) and cut-off values were displayed in **Table 2**. ROC curves showed that the ratios of Lymphocyte, T cell, CD4^+^ T cell, CD8^+^ T cell, and B cell counts after the first treatment cycle to baseline were good predictors of four-cycle treatment efficacy. B cell had the best predictive ability with AUCs of 0.857 (**Figure 3A**, *P* = 0.002) and 0.856 (**Figure 3B**, *P* < 0.001) for patients receiving chemotherapy and combination immunotherapy, respectively. Patients with NSCLC who received the first cycle of chemotherapy or combination immunotherapy had a peripheral blood B cell count to baseline ratio greater than 0.825 or 0.93, respectively, indicating that they were most likely to have good tumor control after four cycles of regular treatment. The AUCs for Lymphocyte, T cell, CD4^+^ T cell and CD8^+^ T cell were all greater than 0.75, indicating their predictive ability (P < 0.002). Based on these data, only NK cell was not an efficacy predictor (**Figure 3A**, P = 0.109, **Figure 3B**, P = 0.238). In addition, we attempted to construct models based on combined lymphocyte subsets through logistic regression for higher predictive power. Since there were only 21 non-response patients in this study, according to the rule of 10 events per variable in logistic model, we considered synthesizing two lymphocyte subsets in order to jointly predict efficacy. And we excluded total lymphocytes from the logistic regression due to the collinearity and correlation problems. By performing regression analyses on combinations of different two lymphocyte subsets, we determined that combinations of T cell plus B cell (AUC=0.88, P<0.001) and CD8^+^ T cell plus B cell (AUC=0.878, P<0.001) showed excellent predictive power and were better than single lymphocyte subset. Regression analyses and forest plots were demonstrated in **Figure 3C**. And the reliability and accuracy of the predictive models were evaluated.

**Table 2:** Predictive ability of lymphocyte subsets after the first treatment cycle.

	Variable	AUC	Asymptotic significance	Asymptotic 95% Confidence		Cut-off Value
				Lower Bound	Upper Bound	
Chemotherapy	Lymphocyte	0.789	0.001	0.612	0.966	0.93
	T cell	0.811	< 0.001	0.644	0.977	0.86
	CD4^+^ T cell	0.831	< 0.001	0.677	0.985	0.92
	CD8^+^ T cell	0.783	0.002	0.602	0.964	0.855
	B cell	0.857	< 0.001	0.714	1.001	0.825
	NK cell	0.657	0.109	0.465	0.848	
Combination	Lymphocyte	0.818	0.001	0.631	1.004	0.99
	T cell	0.822	< 0.001	0.644	1.000	0.895
	CD4^+^ T cell	0.811	0.001	0.620	1.002	0.8
	CD8^+^ T cell	0.831	< 0.001	0.677	0.985	0.955
	B cell	0.856	< 0.001	0.725	0.986	0.93
	NK cell	0.631	0.238	0.413	0.849	

The predictive ability of each lymphocyte subset for efficacy was analyzed using ROC curves and asymptotic significance at P < 0.05 was considered statistically significant.

### 3.3 Combination immunotherapy improved lymphopenia caused by chemotherapy toxicity

**Figure 4:**
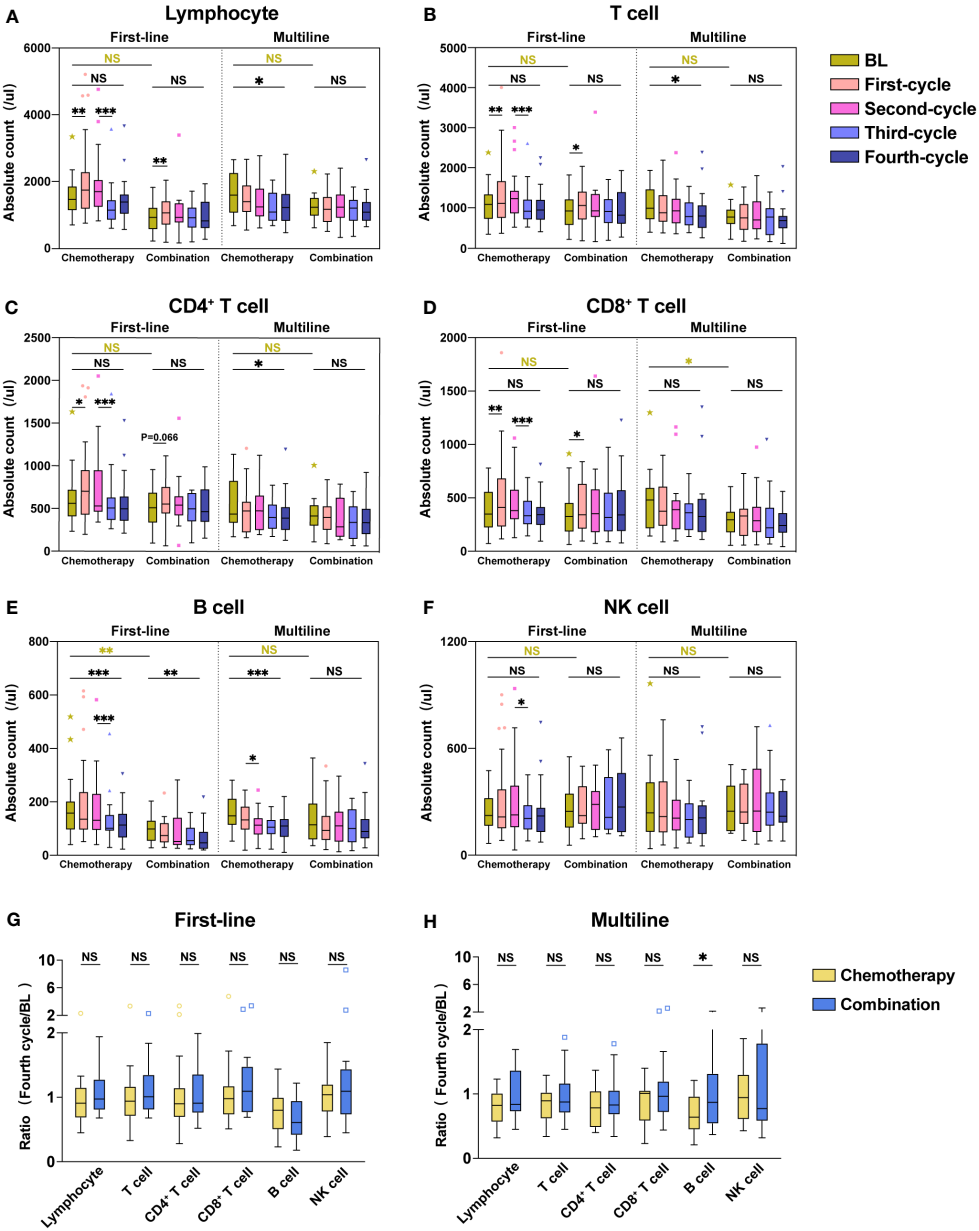
Combination immunotherapy improved absolute counts of lymphocyte subsets compared with chemotherapy alone. Absolute counts of Lymphocyte **(A)**, T cell **(B)**, CD4^+^ T cell **(C)**, CD8^+^ T cell **(D)**, B cell **(E)**, and NK cell **(F)** in the first-line group and multiline group during four consecutive treatment cycles were shown as boxplots. The ratios of each lymphocyte subset after four treatment cycles to baseline in the first-line **(G)** and multiline **(H)** patients. The chemotherapy and combination immunotherapy groups were shown, respectively. Within-group and between-group differences were analyzed using the paired Wilcoxon signed-rank test and Mann-Whitney U test, respectively. BL, Baseline; *, P < 0.05; **, P < 0.01; ***, P < 0.001; NS, not significant.

In our study, each lymphocyte subset showed a significant reduction after the third cycle of chemotherapy, while this lymphopenia was significantly ameliorated with combination immunotherapy. We found that combination immunotherapy protected against decreased lymphocyte and increased the lymphocyte counts. To confirm our hypothesis, we analyzed each lymphocyte subset in the chemotherapy and combination immunotherapy groups (**Figure 4**). To exclude baseline differences due to previous treatment, first-line and multiline patients were analyzed respectively. Among patients receiving first-line treatment, both the chemotherapy and combination groups showed significant increases in Lymphocyte (**Figure 4A**), T cell (**Figure 4B**), CD4^+^ T cell (**Figure 4C**), and CD8^+^ T cell (**Figure 4D**) counts after the first treatment. The chemotherapy group showed a significant reduction in all lymphocyte subsets after the third treatment cycle, whereas no significant reduction was observed in the combination immunotherapy group (**Figures 4A–F**). Except for B cell (**Figure 4E**), there were no significant differences in lymphocytes counts before and after four cycles of treatment in first-line chemotherapy patients. Observably, lymphocyte subsets were significantly maintained at relatively higher levels in the combination immunotherapy group. In the multiline group, Lymphocyte (**Figure 4A**), T cell (**Figure 4B**), CD4+ T cell (**Figure 4C**), CD8+ T cell (**Figure 4D**), and B cell (**Figure 4E**) were significantly decreased after four chemotherapy cycles, whereas immunotherapy maintained lymphocytes at relatively high levels. There were no significant differences in the lymphocyte subsets before and after the four cycles of combination therapy. We further analyzed the ratios of each lymphocyte subset after four treatment cycles to baseline (**Figures 4G, H**). Lymphocyte subsets in the combination immunotherapy group demonstrated relatively higher levels overall in both the first-line and multiline patients, although the ratios between the chemotherapy and combination groups were not statistically significant, with only B cell in the multiline group exhibiting a significant increase following combination immunotherapy (**Figure 4H**, *P* = 0.034).

### 3.4 Significant differences in dynamic changes of lymphocyte subsets in the first-line and multiline treatments

**Figure 5:**
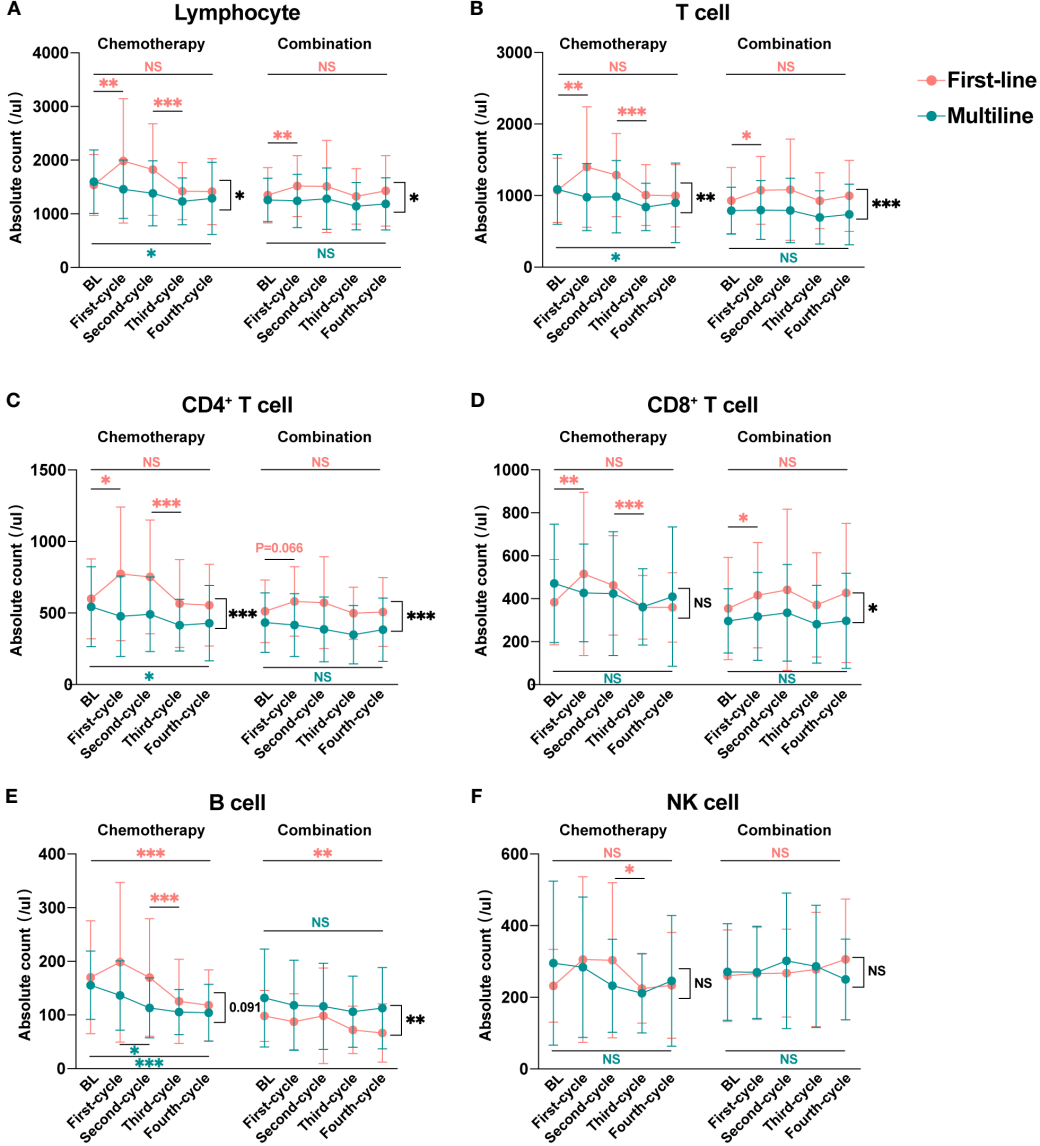
Dynamic changes in lymphocyte subsets during four treatment cycles in the first-line and multiline groups. Median absolute counts and interquartile ranges of Lymphocyte **(A)**, T cell **(B)**, CD4^+^ T cell **(C)**, CD8^+^ T cell **(D)**, B cell **(E)**, and NK cell **(F)** in the first-line group and multiline group during four consecutive treatment cycles were shown as folded line charts. The chemotherapy and combination immunotherapy groups were shown, respectively. Within-group differences and between-group differences were analyzed using the paired Wilcoxon signed-rank test and Mann-Whitney U test, respectively. BL, Baseline; *, P < 0.05; **, P < 0.01; ***, P < 0.001; NS, not significant.

According to our inclusion criteria, 45 patients in the first-line group were treated for the first time after diagnosis, and 36 patients in the multiline group were treated again after at least second-line failure. To explore the dynamic changes in lymphocyte subsets in patients receiving first-line and multiline treatments during chemotherapy or combination immunotherapy, within-group and between-group differences were analyzed, and different treatments were displayed respectively (**Figure 5**).

Lymphocyte (**Figure 5A**), especially T cell (**Figure 5B**), including CD4^+^ T cell (**Figure 5C**) and CD8^+^ T cell (**Figure 5D**), were significantly increased after the first cycle of chemotherapy or combination immunotherapy in the first-line group. Lymphocyte subsets began to decrease after the second treatment cycle, with a significant decrease after the third chemotherapy cycle. Those decreases were more significant in the chemotherapy group. Changes in lymphocyte subsets were unsatisfactory in the multiline group, with a rapid decrease, whereas combination immunotherapy significantly improved this problem. NK cell (**Figure 5F**) showed a similar trend, however, owing to the small number of cases included in this study and the large individual differences, no statistical differences were observed. However, abnormal behavior was observed for B cell (**Figure 5E**), which decreased significantly in the first-line patients who received combination therapy, but no significant decrease was observed in the multiline patients.

## 4 Discussion

PD-1 inhibitor in combination with chemotherapy has been the first-line standard treatment in advanced NSCLC. Although immunotherapy has pronounced excellent results, it is undeniable that some patients are insensitive to the treatment, resulting in a failure to benefit from it, which emphasizes the importance of early efficacy prediction in tumor treatment (23, 24). This prospective study aimed to analyze the differences in lymphocyte subsets with different efficacies to determine the potential predictive power of lymphocyte subsets. Our results showed that the counts of lymphocyte subsets in the response group significantly increased, in contrast to a rapid decline in the non-response group. Simultaneously, we found that patients who received anti-PD-1 based immunotherapy had higher lymphocyte levels relative to chemotherapy alone. We also noted the significant differences in the lymphocytes counts between patients who received first-line and multiline treatments.

Lymphocytes, including three major subsets of T, B, and NK cells, are the main executors of the adaptive immune system and play vital roles in tumor control through surveillance and destruction (25). T cells, with the CD3 as the surface marker, are divided into helper T cells, marked by the CD4 molecule and cytotoxic T cells, marked by the CD8 molecule (26). CD8^+^ T cells, the mainstay of adaptive immunity, can infiltrate tumor centers and directly target and kill tumor cells via cytotoxicity (27). Immunotherapy, especially chimeric antigen receptor T (CAR-T) cell therapy based on CD8^+^ T cell, has shown excellent antitumor effects in many types of tumors owing to its targeting and durability (28–30). CD4^+^ T cells are mainly considered as helper cells for the activation of CD8^+^ T cells (31). They can also kill tumor cells directly or indirectly by secreting a variety of cytokines (32). A recent study has reported a new function of CD4^+^ T cells for the first time, in which one specific subtype of CD4^+^ T cells kills tumor cells that escape CD8^+^ T cell attack (33). This suggests the potential to develop CD4^+^ T cells as immunotherapy targets in the future, especially for the patients with cancer who have failed to respond to CD8^+^ T cell therapy. B cells, with the CD19 as surface marker, mainly secrete antibodies against tumor-associated antigens and coactivate CD8^+^ T cells in conjunction with CD4^+^ T cells (34). However, studies have also reported that B cell infiltration in tumors is associated with poor prognosis (35, 36). NK cells are characterized by the surface molecule CD56. NK cells serve as the crucial first line of defense against tumors and pathogens (37). Their cytotoxic and immunomodulatory effects on the tumor microenvironment cannot be ignored (38). NK cell-based tumor immunotherapies have also been explored currently (39, 40). Lymphocytes, as multifunctional biomarkers, have been reported to be valuable for evaluating patient immunity and predicting outcomes (41–43). During chemotherapy, multiple substances released from tumor cells contribute to the activation and proliferation of lymphocytes, working together to kill tumors (44, 45). However, large numbers of inactive or “bystander” lymphocytes in the tumor immune microenvironment will compromise therapeutic efficacy (46). Thus, an assessment of the initial lymphocyte count merely may not accurately reflect the actual immune capacity. In this study, we respectively analyzed the dynamic changes in lymphocyte subsets in the peripheral blood of patients with NSCLC who received four consecutive cycles of chemotherapy or combination immunotherapy with ani-PD-1 antibody. We found the differences in lymphocyte subsets with different efficacies. Based on this, we propose that the ratios of lymphocyte absolute counts after the first chemotherapy or combination immunotherapy cycle to baseline are early and accurate predictors of efficacy.

The early prediction of clinical efficacy is an urgent problem for achieving precise and individualized treatment (47, 48). Early identification of patients with poor outcomes helps adjust treatment plans in a timely manner to improve treatment effects in the clinic, which is of great significance in prolonging the progression-free survival and overall survival of cancer patients. In our study, the response and non-response groups, which showed no significant differences at baseline, exhibited extremely different performances after the first treatment. In contrast to the rapid decline observed in the non-response group, the absolute counts of lymphocyte subsets in the response group exhibited a marked increase. After four cycles of chemotherapy, all lymphocyte subsets in the non-response group were significantly reduced. ROC curve analysis showed that the ratios of absolute lymphocyte count after the first treatment cycle to baseline were good predictors of four-cycle treatment efficacy, except for NK cell. In addition, the combination of T cell and B cell or the combination of CD8^+^ T cell and B cell had a better predictive power which provided a reference for timely identification of insensitive patients and the early prediction of outcomes in clinical practice. Although the counts of lymphocyte subsets at baseline in the non-response group were similar to those in the response group, there may be a higher proportion of anergic or bystander lymphocytes or even severe myelosuppression and an immunosuppressive microenvironment preventing lymphocyte activation and proliferation, which affects their ability towards tumor control (49). Our study highlights the importance of the dynamic detection of lymphocyte subsets in patients with cancer. Assessment of the initial immune environment alone cannot accurately predict treatment outcomes, and a dynamic assessment of the lymphocyte response during treatment may better represent immune function and predict the efficacy more reasonably.

In the response group, the absolute counts of each lymphocyte subset in the chemotherapy group first increased and then decreased, while in the combination group, they were maintained at a high level. We propose that chemotherapy combined with immunotherapy has a protective effect on lymphocytes and ameliorates the lymphopenia caused by prolonged chemotherapy. By analyzing all cases, we found that the persistent combination of anti-PD-1 therapy improved lymphocyte levels in patients receiving first-line or multiline therapy. This study provides a theoretical basis for early combination immunotherapy. On the one hand, lymphocytes in the early chemotherapy stage are in a state of massive proliferation, and high levels of lymphocytes allow anti-PD-1 antibody to work more effectively. On the other hand, combination immunotherapy is able to increase the count of lymphocytes and improve the activity of the tumor immune environment. Combined immunotherapy can provide tumor patients with greater benefits, and chemotherapy in combination with anti-PD-1/PD-L1 therapy has been included in Grade I recommendations for certain NSCLCs (50).

Finally, we found that lymphocyte performance in the multiline treatment group was unsatisfactory. Lymphocyte, especially T cell, were significantly reduced in the multiline group after reaccepting chemotherapy. Persistent lymphocyte count decrease may partly explain their poor efficacy in comparison to the favorable lymphocyte response in the first-line treatment group. This also indicates that lymphopenia may be involved in the resistance to tumor therapy. Severe myelosuppression after multiple chemotherapy treatments leads to a hypoactive immune environment, in which vulnerable lymphocytes are highly susceptible to chemotherapy toxicity, resulting in a rapid decrease in their numbers without a timely replenishment. Therefore, patients with low lymphoid levels or multiple chemotherapy treatments are recommended to be treated with immunostimulants, such as thymopeptides or placental polypeptides. Regularly evaluating the immunity level and improving immunity can collaborate with oncological treatments to achieve greater benefits for the patients. Of course, the abnormal performances of B cell after first-line and multiline treatments have also attracted attention. B cell, the smallest lymphocyte subset among three major subsets, accounts for approximately 10% of all lymphocytes (51). Both detection errors and individual differences significantly impacted on the analysis results. Therefore, the abnormality observed in this study was due to errors or unexplored mechanisms requiring multicenter large-sample data or scientific experiments for further verification.

However, this study has some limitations. Only 81 cases were included in this retrospective study. The small sample size was due to the impact of COVID-19 in recent years, which made it difficult to collect complete data covering four consecutive cycles. Factors, such as local treatment and loss to follow-up, influenced data collection. Besides, only the absolute counts of lymphocyte subsets in patients were analyzed in this study. The functions of lymphocytes and other complex tumor microenvironment components have not been considered. In the future, we plan to collect more cases and conduct prospective studies. Long-term dynamic monitoring of lymphocyte subsets in chemotherapy patients, not just limited to four cycles, will allows for a better prediction of efficacy. Importantly, in the future, we expect to build integrated models that combine immune, tumor, and personal characteristics to predict treatment efficacy more accurately.

Currently, the detection of absolute lymphocyte subset counts is largely limited to infectious and immunological diseases in practical clinic application. With the development of the immune surveillance theory, the efficacy of immunotherapy is directly affected by lymphocytes, suggesting a great application space in the field of cancer. Our study provides a reference for the prediction of tumor efficacy and confirms that this simple and easy clinical test can evaluate the real immune status, which is valuable for the timely application of immunostimulants or early replacement of insensitive chemotherapy regimens in clinical treatment. With the accumulation of relevant evidence, the detection of lymphocyte subsets will surely play an important role in the field of oncology. It is reasonable to expect that the rapid and effective detection of peripheral blood lymphocyte subsets will contribute to non-invasive early screening and accurate prognosis of cancer. The realization of this goal is of great significance for the survival of patients with cancer. However, more comprehensive clinical data are yet to be generated by large-scale clinical testing.

## 5 Conclusions

In this study, we identified an association between lymphocyte subsets and the prognosis of patients with NSCLC, which may contribute to the early prediction of the efficacy during chemotherapy or combination immunotherapy. Combination anti-PD-1 therapy protected the immune microenvironment and increased the lymphocyte counts. Patients receiving multiline treatment showed a rapid decrease in lymphocytes, which may be related to the poor efficacy. In summary, dynamic surveillance of lymphocyte subsets allows for the effective assessment of the immune status and the prediction of outcomes in patients with NSCLC.

## Data availability statement

The original contributions presented in the study are included in the article/supplementary material. Further inquiries can be directed to the corresponding authors.

## Ethics statement

The studies involving humans were approved by medical ethics committee of the First Affiliated Hospital of Zhengzhou University. The studies were conducted in accordance with the local legislation and institutional requirements. Written informed consent for participation was not required from the participants or the participants’ legal guardians/next of kin in accordance with the national legislation and institutional requirements.

## Author contributions

SZ: Data curation, Formal analysis, Writing – original draft. WW: Data curation, Formal analysis, Writing – original draft. GQ: Funding acquisition, Investigation, Writing – review & editing. TL: Supervision, Writing – review & editing. LY: Supervision, Writing – review & editing. YZ: Supervision, Writing – review & editing.
